# A Novel Natural Influenza A H1N1 Virus Neuraminidase Inhibitory Peptide Derived from Cod Skin Hydrolysates and Its Antiviral Mechanism

**DOI:** 10.3390/md16100377

**Published:** 2018-10-10

**Authors:** Jianpeng Li, Yiping Chen, Ning Yuan, Mingyong Zeng, Yuanhui Zhao, Rilei Yu, Zunying Liu, Haohao Wu, Shiyuan Dong

**Affiliations:** 1 College of Food Science and Engineering, Ocean University of China, Qingdao 266003, China; changjing@stu.ouc.edu.cn (J.L.); chenyiping@stu.ouc.edu.cn (Y.C.); ningy1@126.com (N.Y.); liuzunying@ouc.edu.cn (Z.L.); wuhaohao@ouc.edu.cn (H.W.) dongshiyuan@ouc.edu.cn (S.D.); 2 School of Medicine and Pharmacy, Ocean University of China, Qingdao 266003, China; ryu@ouc.edu.cn

**Keywords:** cod skin, NA-inhibitory peptide, influenza virus, neuraminidase, molecular docking, adsorption

## Abstract

In this paper, a novel natural influenza A H1N1 virus neuraminidase (NA) inhibitory peptide derived from cod skin hydrolysates was purified and its antiviral mechanism was explored. From the hydrolysates, novel efficient NA-inhibitory peptides were purified by a sequential approach utilizing an ultrafiltration membrane (5000 Da), sephadex G-15 gel column and reverse-phase high-performance liquid chromatography (RP-HPLC). The amino acid sequence of the pure peptide was determined by electrospray ionization Fourier transform ion cyclotron resonance mass spectrometry (ESI-FTICR-MS) was PGEKGPSGEAGTAGPPGTPGPQGL, with a molecular weight of 2163 Da. The analysis of the Lineweacer–Burk model indicated that the peptide was a competitive NA inhibitor with Ki of 0.29 mM and could directly bind free enzymes. In addition, docking studies suggested that hydrogen binding might be the driving force for the binding affinity of PGEKGPSGEAGTAGPPGTPGPQGL to NA. The cytopathic effect reduction assay showed that the peptide PGEKGPSGEAGTAGPPGTPGPQGL protected Madin–Darby canine kidney (MDCK) cells from viral infection and reduced the viral production in a dose-dependent manner. The EC_50_ value was 471 ± 12 μg/mL against H1N1. Time-course analysis showed that PGEKGPSGEAGTAGPPGTPGPQGL inhibited influenza virus in the early stage of the infectious cycle. The virus titers assay indicated that the NA-inhibitory peptide PGEKGPSGEAGTAGPPGTPGPQGL could directly affect the virus toxicity and adsorption by host cells, further proving that the peptide had an anti-viral effect with multiple target sites. The activity of NA-inhibitory peptide was almost inactivated during the simulated in vitro gastrointestinal digestion, suggesting that oral administration is not recommended. The peptide PGEKGPSGEAGTAGPPGTPGPQGL acts as a neuraminidase blocker to inhibit influenza A virus in MDCK cells. Thus, the peptide PGEKGPSGEAGTAGPPGTPGPQGL has potential utility in the treatment of the influenza virus infection.

## 1. Introduction

The influenza virus remains a highly contagious pathogen that causes high morbidity and mortality [1]. In 2009, the influenza A (H1N1) virus first emerged and resulted in more than 8000 deaths worldwide [2]. Furthermore, the number of patients suffering from influenza continues to grow. Neuraminidase (NA), a surface glycoprotein, is a major structural component of the virion and plays an important role in virus replication [3]. Therefore, it is essential to explore the inhibition of NA in order to control the influenza virus.

NA inhibitors can bind to the active site of the viral NA to interfere with the virus replication [1] and are a promising target for screening anti-influenza drugs. Current NA inhibitor drugs, such as zanamivir and oseltamivir [4,5], significantly affect the duration of infection and clinical diseases [6]. However, the pharmaceutical drug efficacy, resistance and cost remain to be solved [7]. In addition, the NA inhibitor drugs may lead to some side effects, such as potential neurotoxicity, digestive discomfort and respiratory diseases [8]. Hence, it is necessary to develop alternative and natural NA inhibitors. Recently, many NA-inhibitory peptides have been found to show potential as antiviral drugs. For example, Amri et al. [9] found that some cyclic peptides, RRR and RRP, showed high NA-inhibitory activity. In addition, Upadhyay et al. [10] found that the mimosine tetrapeptide (M-FFY) also showed high NA-inhibitory activity. However, natural NA-inhibitory peptide was seldom reported.

Cod is an important fish species in China. Lots of scraps are generated during the processing and utilization of cod, which can lead to the waste of resources and environmental pollution. Thus, it is necessary to make full use of the cod scraps. More than 80% of the dry matter of cod skin [11] is collagen, which is rich in proline and l-hydroxyproline [12]. Both proline and l-hydroxyproline contain a pyrrolidine structure [13] and this group can act as hydrogen bond donors or receptors to exert anti-influenza effects by binding to neuraminidase, thus allowing the formation of proline-containing polypeptide inhibitors. A number of studies have showed that synthetic pyrrolidine-containing compounds exhibited high NA-inhibitory activity [14,15], which suggested that natural pyrrolidine-containing substances (proline and l-hydroxyproline) might also play an important role in the inhibition of NA. Recently, cod skins were widely applied in the preparation of ACE-inhibitory peptides [16,17]. However, the preparation of natural NA-inhibitory peptides from cod skin hydrolysates was not reported. Therefore, the peptide derived from cod skin hydrolysates might have the high potential in the inhibition of NA. In addition, it has been reported that the influenza-infection cycle involves several distinct steps [5,18]. Hemagglutinin (HA), a significant surface glycoprotein, also plays an important role in viral infection by mediating viral entry and fusion [19,20,21]. Thus, it is significant to further investigate the potential mechanisms of peptides against the influenza A virus.

To the best of our knowledge, the preparation of natural influenza A H1N1 virus neuraminidase inhibitory peptide from cod skin hydrolysates has seldom been reported. The study aims to prepare efficient NA-inhibitory peptides from cod skin hydrolysates. We identified the NA-inhibitory peptides with high activities by electrospray ionization Fourier transform ion cyclotron resonance mass spectrometry (ESI-FTICR-MS). In addition, molecular docking simulations were conducted to investigate the interactions between the peptides and NA. Moreover, the mechanism of the peptide against the influenza virus was also discussed. This study can provide previously unknown information about the effect of the novel NA-inhibitory peptides on influenza A H1N1 virus and alternative approach for antiviral therapy.

## 2. Results and Discussion

### 2.1. Isolation and Purification of Neuraminidase (NA)-Inhibitory Peptide

**Figure 1:**
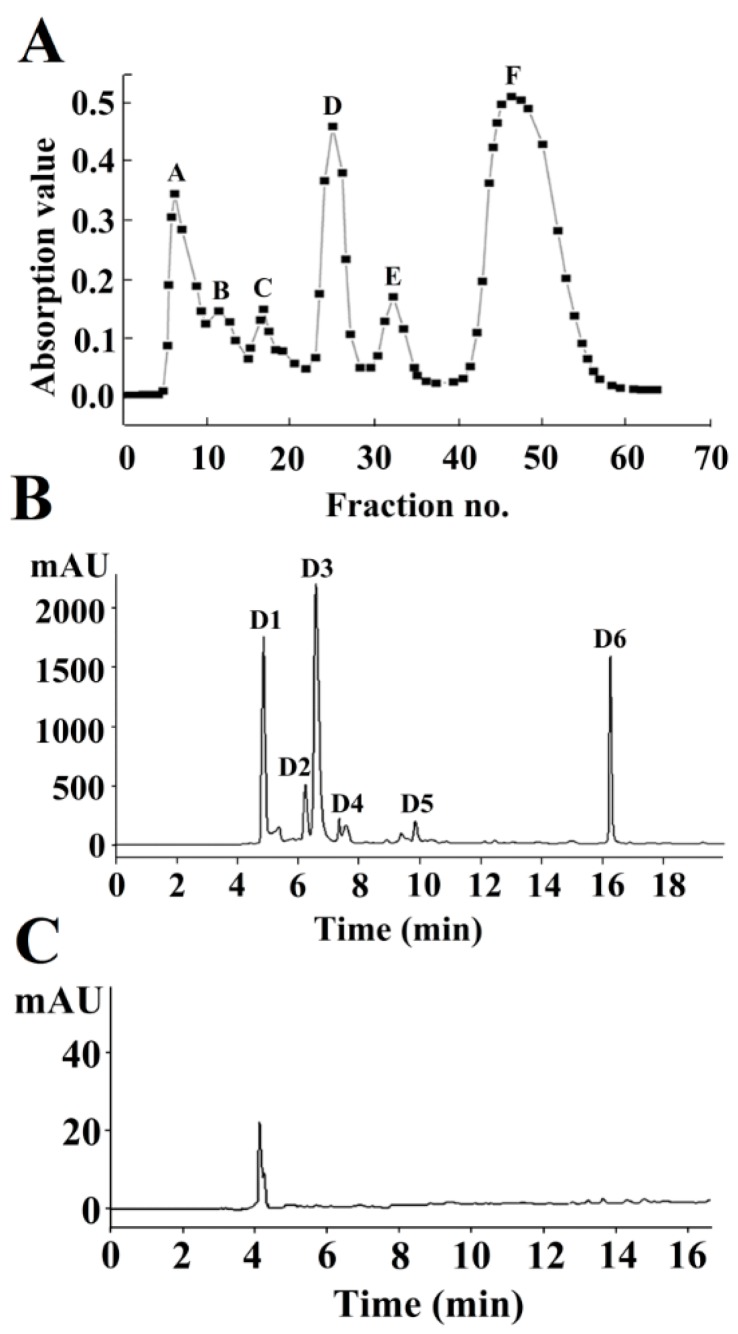
Isolation and purification of NA-inhibitory peptides using a Sephadex G-15 gel column (**A**) and reverse-phase high-performance liquid chromatography (RP-HPLC) (**B**). Purity identification of neuraminidase (NA)-inhibitory peptide D1 (**C**).

The enzymatic hydrolysates of cod skins were firstly ultrafiltered with an 5 K membrane to obtain the components whose molecular weight were less than 5000 Da. The IC_50_ value of the ultrafiltrate was 6.4 mg/mL (Table 1). The NA-inhibitory peptides were then fractionated using a Sephadex G-15 gel column and Fractions A–F were obtained at 220 nm (Figure 1A). The NA inhibition assays of these fractions showed that Fraction D exhibited high NA-inhibitory activity (IC_50_ = 3.50 ± 0.11 mg/mL). The fractions with the same molecular weight peaked simultaneously in the Sephadex G-15 gel column [22]. Therefore, Fraction D may be a mixture and needs to be further fractionated by reverse-phase high-performance liquid chromatography (RP-HPLC).

RP-HPLC is a common tool for isolating and purifying the polypeptides [22]. After 5 min of elution, six major peaks were detected at 220 nm, among which the peak corresponding to Fraction D1 exhibited a relatively high intensity (Figure 1B). Fraction D1 exhibited the high activity (IC_50_ = 0.89 ± 0.07 mg/mL). After a two-step purification process, Fraction D1 was purified by 7.19 times (Table 1), suggesting that the NA-inhibitory activity of cod skin peptides can be significantly improved by fractionation and purification. In addition, Fraction D1 exhibited a single peak in an analytical C_18_ HPLC column (Figure 1C), suggesting that the purity of D1 had met the requirement for sequencing.

### 2.2. Identification of the NA-Inhibitory Peptide

**Figure 2:**
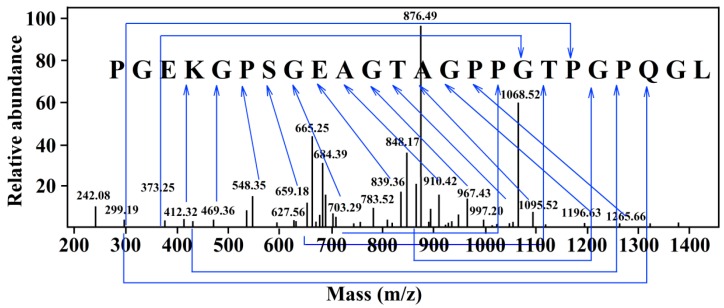
Electrospray ionization Fourier transform ion cyclotron resonance mass spectrometry (ESI-FTICR-MS) spectra of the amino acid sequences of Fraction D1.

ESI-FTICR-MS can simultaneously dissociate multiple precursor ions and has a wide detection range, high resolution, and high precision [23]. To determine the matching degree of the identification sequence, the sequence results were matched by using the Swiss Prot database. The matching result showed that the determined sequence was PGEKGPSGEAGTAGPPGTPGPQGL with a molecular mass of 2163 Da (Figure 2). The peptide consisted of 24 amino acid residues and proline accounted for a quarter.

### 2.3. Mode of Action and Molecular Docking of PGEKGPSGEAGTAGPPGTPGPQGL

**Figure 3:**
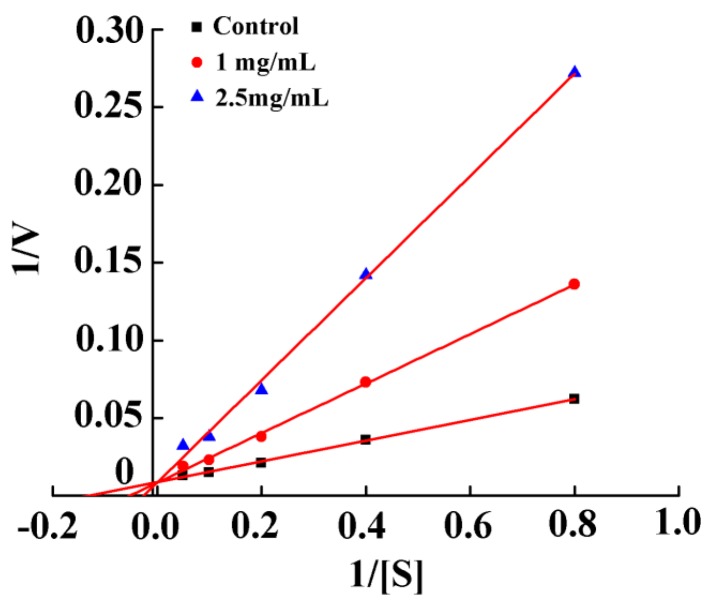
Kinetic study of the NA inhibition profile of PGEKGPSGEAGTAGPPGTPGPQGL.

To determine the mode of action of the NA-inhibitory peptide, a Lineweaver–Burk kinetic model was used to explore the relationship between the reaction rate and the substrate concentration. As shown in Figure 3, PGEKGPSGEAGTAGPPGTPGPQGL (peptide P) is a competitive NA inhibitor (Ki = 0.29 mM), suggesting that the peptide P can directly bind free enzyme. Such a binding results in a decrease in substrate affinity at the active site [24]. The binding of NA to a substrate or competitive inhibitor of amino acid residues is the highly specific binding [25]. A number of studies have shown that modes of action of NA inhibitors include competitive, non-competitive, uncompetitive and mixed modes. For example, Park et al. [26] obtained 2-hydroxy-3-methyl-3-butenyl alkyl (HMB) from *Angelica keiskei* and HMB was a non-competitive inhibitor with Ki of 14.0 ± 1.5 μM (IC_50_ = 12.3 μM). Nguyen et al. [27] isolated eight oligostilbenes from *Vitis amurensis*, which were all non-competitive inhibitors with Ki of 8–25 μM (IC_50_ = 8.94–234.61 μM). Jiang et al. [28] isolated indole alkaloid from *Streptomyces* sp. FIM090041, which was a competitive inhibitor with Ki of 13.5 μM (IC_50_ = 67.8 μM). In addition, oseltamivir was a competitive inhibitor of chemically synthesized drugs. Compared with those NA inhibitors, the NA-inhibitory intensity of the peptide P was relatively low. Thus, the synergistic combination with other inhibitors should be further explored based on this study.

**Figure 4:**
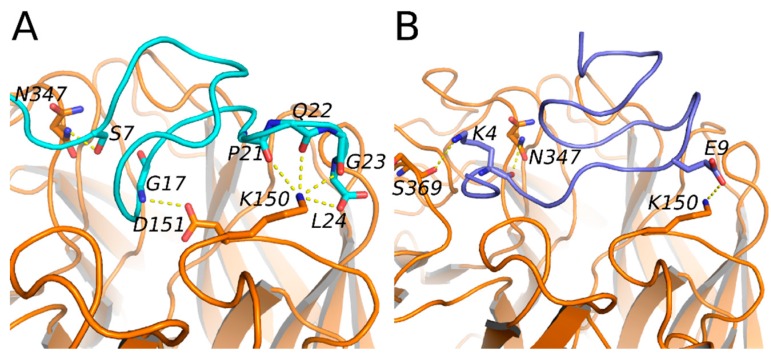
Probable binding mode of peptide PGEKGPSGEAGTAGPPGTPGPQGL at H1N1 NA. Conformation of the peptide was determined by MD simulations. Conformation of the peptide was clustered from the last 50 ns MD. (**A**,**B**) show the binding modes of the two minimum-energy conformations (Conformation **A** and Conformation **B**) of the peptide extracted from the two largest clusters at the binding site of NA. The dashed lines show the hydrogen bonds formed between residues from the peptide and residues of the NA.

For the purpose of understanding the interaction mechanism between peptide P and NA, molecular docking was performed. Prior to docking, the conformation of the peptide P was determined using molecular dynamics (MD). Peptide P has several conformations in the solution. Two main clusters were identified. Two minimum-energy conformations of peptide P from the two main clusters were selected for binding modes determination. As shown in Figure 4, Conformation A forms 6 hydrogen bonds with the residues Asn347, Asp151 and Lys150, whereas Conformation B only forms 3 hydrogen bonds with the residues Lys150, Asn347 and Ser369. In addition, Pro21 of Conformation A is important in the formation of hydrogen bonds. Thus, Conformation A is probably more energetically favorable to bind to the NA. Our docking studies suggested that hydrogen binding might be the driving force for the binding affinity of peptide P to NA. Li et al. [22] also reported that the hydrogen binding was the main driving force for the binding affinity of PNVA to angiotensin converting enzyme (ACE). The binding site of the NA is filled with charged residues and properly introducing charged residues to the NA can be a way to increase the binding affinity of peptide P analogues.

### 2.4. Cytotoxicity and Antiviral Activity of Peptide PGEKGPSGEAGTAGPPGTPGPQGL on Madin–Darby Canine Kidney (MDCK) Cells

**Figure 5:**
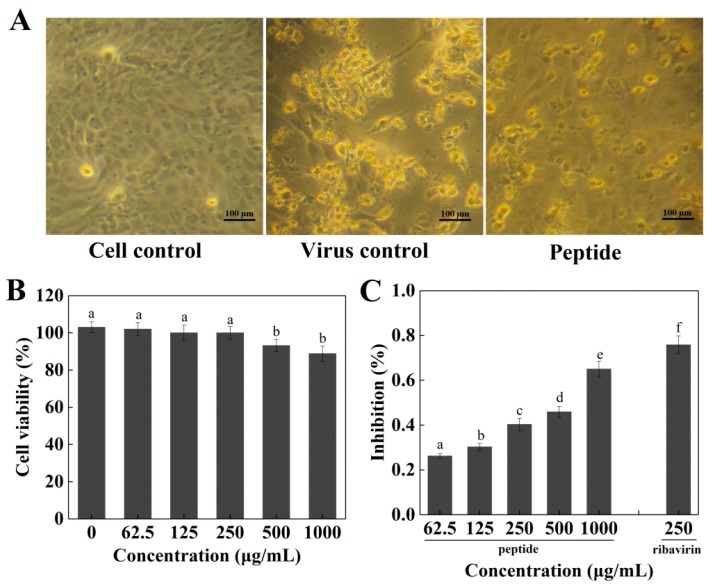
(**A**) Morphological changes in H1N1-infected MDCK cells; (**B**) Cell viability of Madin–Darby canine kidney (MDCK) cells after being incubated with the peptide at different concentrations for 48 h; (**C**) Inhibitory effects of the peptide on influenza virus H1N1 infection in MDCK cells. Values of three replicates are expressed as mean ± standard deviation. Different lowercase letters indicate significantly different values (*p* < 0.05).

The peptide P exhibited no significant cytotoxicity in Madin–Darby canine kidney (MDCK) cells at a concentration of 250 μg/mL or less (*p* > 0.05), while peptide P significantly reduced the viability of MDCK cells at concentrations over 250 μg/mL (*p* < 0.05) (Figure 5B). The inhibitory effect of the peptide P on the H1N1 virus was examined in vitro. MDCK cells treated with H1N1 (Figure 5A) exhibited cytoplasmic shrinkage, loss of cell-cell contract, and reduction in cell numbers. After co-treatment with peptide P, MDCK cell morphology was changed slightly and appeared healthy with a regular shape compared with the control cells. The treatment with 62.5~1000 μg/mL peptide P significantly reduced the cytopathic effect (CPE) and peptide P increased the viability of virus-infected cells dose-dependently (Figure 5C). The peptide P at a concentration of 250 μg/mL inhibited H1N1 by 42%, which showed a lower antiviral effect than ribavirin (*p* < 0.05). The EC_50_ value of peptide P against H1N1 was 471 ± 12 μg/mL. Test results indicated that peptide P could protect MDCK cells from viral infection and reduced the viral production in a dose-dependent manner.

### 2.5. Inhibitory Effects of Peptide PGEKGPSGEAGTAGPPGTPGPQGL on Different Stages of Viral Replication

**Figure 6:**
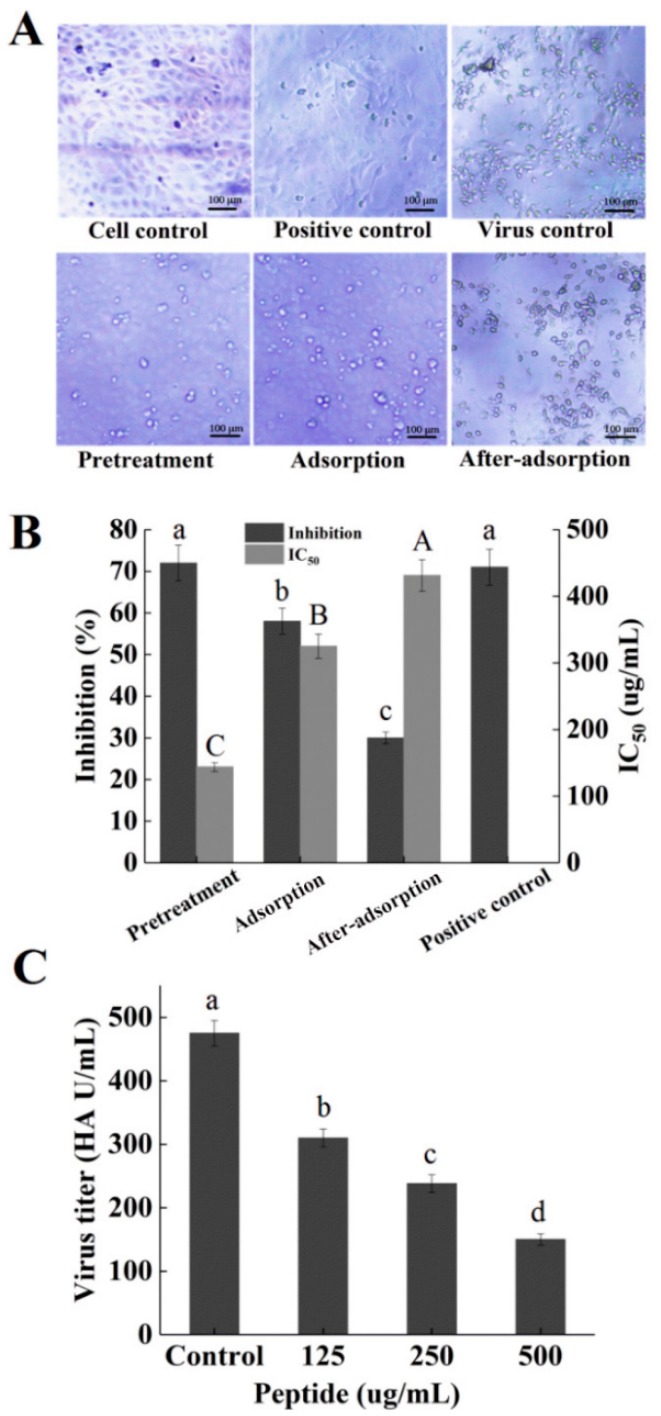
(**A**) Morphological changes of H1N1-infected MDCK cells under different treatment conditions. (i) Pretreatment, (ii) adsorption, (iii) after-adsorption; (**B**) MDCK cells were infected with the peptide under three different treatment conditions; (**C**) hemagglutination (HA) titers of influenza virus H1N1 treated with different concentrations of peptide. Values of three replicates are expressed as mean ± standard deviation. Different letters indicate significantly different values (*p* < 0.05).

To determine the stages in which the peptide P plays a role in the influenza virus life cycle, a time-of-addition experiment was conducted. MDCK cells treated with H1N1 exhibited the shrinkage of cytoplasm, loss of cell–cell contract, and apoptosis (Figure 6A). The MDCK-cell morphology was different in three stages and the pretreatment group appeared healthier than adsorption and after-adsorption groups, with regular shapes. A significant protection effect was observed when the peptide P was added before viral adsorption and the inhibition ratio was 73%, which was slightly lower than the positive control of ribavirin (75%) (Figure 6B), suggesting that the possible target of peptide P was located in the cell surface and could reduce viral virulence. In addition, the viability of the infected cells was partly recovered by the peptide P during viral adsorption, and the inhibition ratio was 58%, which was significantly lower than positive control of ribavirin (75%) (Figure 6B), indicating that peptide P could effectively prevent the attachment of virus and cells. Moreover, peptide P showed less inhibition rates by approximately 31.5% against H1N1 when it was added after the infection (Figure 6B), indicating that peptide P could inhibit an after-adsorption step of the influenza virus life cycle. In addition, the IC_50_ values in three different stages of viral replication were increased according to the following order: pretreatment < adsorption < after-adsorption (Figure 6B). The result was consistent with the results of inhibition assays. Overall, the time-course analysis showed that the peptide P mainly inhibited influenza virus in the early stage of the infectious cycle.

Previous studies reported the protein-enriched fraction (PEF) that isolated from the larvae of housefly had strong antiviral activity against influenza virus at a very early stage of the interaction with virus particles or their entry into the cells [29]. The extract of *Ginkgo biloba* (EGB) could directly interact with influenza virus and markedly reduce the infectivity of the virus by preventing the adsorption to host cells [30]. The theaflavin derivatives had a direct effect on viral particle infectivity [31]. Similar to the results in the study, these studies also indicated that the inhibition of influenza virus occurred in the early stage of the infectious cycle. Ding et al. [32] reported that chlorogenic acid (CHA) inhibited influenza virus in the late stage of the infectious cycle.

### 2.6. Hemagglutination (HA) Assay

To explore whether the inhibitory effect of the peptide P on H1N1 was cell-specific or not, the virus titers in MDCK cell supernatants were measured by hemagglutination (HA) assay. The virus particles contain hemagglutinin protein, which binds to receptors on the surface of erythrocytes and causes hemagglutination (HA) [33]. The maximum dilution ratio of red blood cell agglutination can be set as the HA titer in HA experiments with different dilutions of virus. Some active substances can bind to the surface of the virus and block the hemagglutinin, thereby preventing the combination of red blood cells and inhibiting hemagglutination. As shown in Figure 6C, the HA titer of the virus was inhibited by the peptide P dose-dependently and the peptide P could significantly reduce the virus titer at the concentration over 125 μg/mL (*p* < 0.05). The NA-inhibitory peptide P could affect the virus toxicity and adsorption by host cells, further proving that the peptide had an anti-viral effect with multiple target sites. Wang et al. [29] also reported that PEF could decrease the infectious capacity in HA titer and prevent the attachment of virus and cells. In addition, Haruyama et al. [30] reported that EGB contained an anti-influenza virus substance that directly affected influenza virus particles and disrupted the function of hemagglutinin in the adsorption to host cells.

### 2.7. Simulated Digestion Test on NA-Inhibitory Peptide

**Figure 7:**
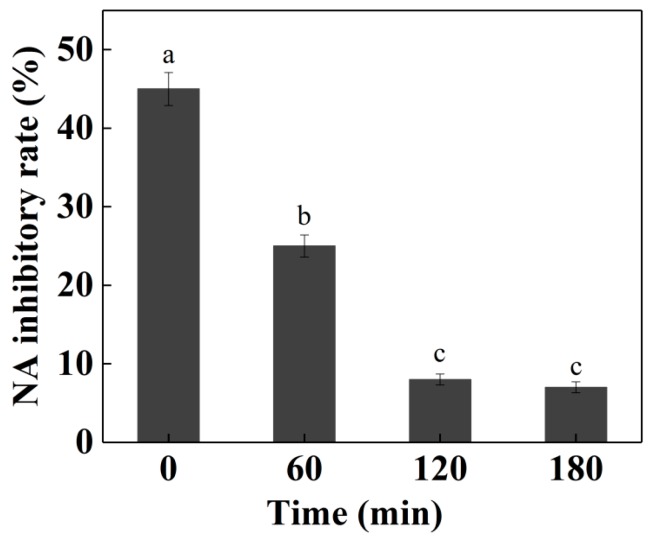
NA-inhibitory rate of the peptide PGEKGPSGEAGTAGPPGTPGPQGL during the simulated in vitro gastrointestinal digestion. Values of three replicates are expressed as mean ± standard deviation. Different lowercase letters indicate significantly different values (*p* < 0.05).

The NA-inhibitory peptides need to resist digestive enzymes in vivo to maintain the stability of the peptides. In the simulated environment of the digestive tract, the activity the NA-inhibitory peptides before and after digestion were measured. To explore the behaviors of the peptide in the digestive tract in vitro, the peptide P was treated with digestive enzymes. After the simulated digestion, the activity of the NA-inhibitory peptide was significantly decreased (*p* < 0.05), indicating the instability of the NA-inhibitory peptide during the simulated in vitro gastrointestinal digestion (Figure 7). Kuba et al. [34] extracted ACE inhibitory peptide Trp-Leu from Tofuyo (fermented soybean food) and suggested that the inhibitory activity of Trp-Leu was completely preserved after the simulated digestion. Different peptides have different tolerances to gastrointestinal proteases. The activities of some functional peptides were increased after digestion and could be taken orally as a prodrug through sustained release or direct digestion. However, the activity of NA-inhibitory peptide in cod skin hydrolyzates after digestion was not ideal, so oral administration is not recommended. There are many ways to uptake the NA-inhibitory peptides, including adsorption, injection, oral administration and other ways. Therefore, it is necessary to further explore other routes of entry.

## 3. Materials and Methods

### 3.1. Reagents

Cod skins were purchased from Shandong Meijia Group Co., Ltd. (Rizhao, China). Ribavirin (50 mg/mL) was purchased from Cisen Pharmaceutical Co., Ltd. (Jining, China). Zorbax SB-C18 column (9.4 mm × 250 mm) was obtained from Agilent Technologies (Santa Clara, CA, USA). RPM1640 medium, fetal bovine serum (FBS), penicillin and streptomycin were obtained from Gibco (Grand Island, NY, USA). 4-Methylumbelliferyl-*N*-acetyl-α-d-neuralminic acid (MUNANA) and 2-(*N*-morpholino) ethanesulphonic acid (MES) were bought from Sigma (St. Louis, MO, USA). Influenza virus H1N1 neuraminidase (NA) was kindly made by the School of Medicine and Pharmacy, Ocean University of China (Qingdao, China). All other chemicals were bought from local commercial sources and were of the highest purity available.

### 3.2. Cells and Viruses

MDCK cells were purchased from Shanghai Institute of Biochemistry and Cell Biology (Shanghai, China), and maintained in RPM1640 medium included 10% of FBS, 100 U/mL of penicillin and 100 μg/mL of streptomycin. The influenza virus A/Puerto Rico/8/1934 (H1N1) was obtained from Wuhan Institute of Virology (Wuhan, China), and grown in 10-day-old embryonated eggs at 36.5 °C for 72 h. As for infection, virus propagation solution was diluted in phosphate buffered saline (PBS) containing 0.2% bovine serum albumin (BSA) and then added into the cells at the indicated multiplicity of infection (MOI). Viruses were allowed to be adsorbed 60 min at 37 °C. After removing the virus inoculum, cells were maintained in infecting media (RPM1640, 4 µg/mL trypsin) at 37 °C in 5% CO_2_.

### 3.3. Protein Extraction of Cod Skins

The protein of cod skins was prepared according to the method described by Zhao et al. [35]. Briefly, 100 g of cod skins were firstly cut up and homogenized in 600 mL of distilled water. Then, protein of cod skins was extracted from the homogenates in a water bath at 85 °C for 6 h and centrifuged (5000× *g*, 30 min) to obtain the solutions. The solutions were freeze-dried with a lyophilizer (Ningbo Scientz Biotechnology Co., Ltd., Ningbo, China).

### 3.4. Preparation of Cod Skin Protein Hydrolysates

The freeze-dried cod skin protein was initially dissolved in water and its substrate concentration was adjusted to 22 mg/mL. After that, pepsin (1.6 kU/g protein) was added into the solution and hydrolyzed in a water bath at 37 °C for 6.8 h. Afterward, the pepsin was inactivated at 100 °C for 10 min. After centrifugation at 5000× *g* for 30 min, the ultrafiltration precipitation was carried out with 5 K membrane (Millipore Isopore, Billerica, MA, USA). The precipitate obtained was freeze-dried and stored for use.

### 3.5. Purification of NA-Inhibitory Peptide

The Sephadex G-15 column (2.6 cm × 65 cm) was used to purify the NA-inhibitory peptides. The flow rate was 1.2 mL/min and double distilled water was used for elution. After that, the Zorbax SB-C18 column (9.4 mm × 250 mm, Agilent, CA, USA) equipped with an Agilent 1260 infinity HPLC system (Agilent Technology, Mississauga, ON, Canada) was used to analyze the peptides in the hydrolysates at a flow rate of 1.5 mL/min. An acetonitrile gradient from 5% to 40% was adopted for 20-min elution to separate groups of peptides. Chromatographic separation was carried out at 35 °C. The components were collected at the absorbance of 220 nm and freeze-dried for further analysis.

### 3.6. NA-Inhibitory Activity Assay

The NA activity assay was performed according to the previous method with slight modifications [36]. Briefly, the sample (10 μL) and NA solution (30 μL) were added to a 96-well plate. After the incubation at 37 °C for 30 min, 60 μL of reaction buffer (33 mM MES buffer, pH = 3.5; 4 mM CaCl_2_; 10 μL MUNANA) was added. After the incubation under the same conditions, 100 μL of stop solution (83% ethanol; 14 mM NaOH) was added to each well. The fluorescence intensity was measured with a SpectraMax M5 plate reader (Molecular Decices, Sunnyvale, CA, USA) with the excitation and emission wavelengths of 355 and 460 nm, respectively; 10 μL of PBS and 30 μL of NA were used as the positive control, and 10 μL of PBS and 30 μL of PBS were used as the negative control. The hydrolysates (10 μL) and NA (30 μL) were used as the samples and 10 μL of hydrolysates and 30 μL of PBS were used as the negative control of the sample. The inhibition activity is calculated as:

(1) $$\mathrm{Inhibition}\;\mathrm{activity}\left(\%\right)=\frac{\left(A_{\mathrm{PC}}-A_{\mathrm{NC}}\right)-\left(A_{\mathrm{Sample}}-A_{\mathrm{SNC}}\right)}{A_{\mathrm{PC}}-A_{\mathrm{NC}}}\times 100\%$$

PC: positive control, NC: negative control, SNC: negative control of the sample.

### 3.7. Amino Acid Sequence Analysis

The peptides were sequenced using ESI-FTICR-MS. The sequencing was completed in the Beijing Proteome Research Center (Beijing, China).

### 3.8. NA Inhibition Mode

The mode of NA inhibition was determined according to the previous method with slight modifications [37]. Various concentrations of MUNANA (1.25, 2.5, 5, 10 and 20 μM) were incubated with NA in the absence or presence (0–2.5 mg/mL) of the peptide P at 37 °C. The inhibition kinetics of NA in the presence of peptide P was determined based on the Lineweaver–Burk plot. The inhibitor constant Ki was calculated by plotting 1/Vmax versus the concentrations of peptide P.

### 3.9. Molecular Dynamics Simulation

The initial conformation of the peptide P was produced with the xleap module in AMBER 16. The protonation states of the peptide P residues were predicted with the PropKa 3.1 method [38]. The initial model was minimized and refined through molecular dynamics (MD) simulations with the Amber 16 package and ff14SB force field [39]. The system was minimized and equilibrated according the previous method [40]. The SHAKE algorithm was used for the MD simulations [41]. The long-range electrostatic interactions were modeled using the particle-mesh ewald (PME) method [42]. MD trajectories were analyzed with VMD [43] and molecules were drawn with PyMol (Schrödinger LLC, Portland, OR, USA).

### 3.10. Docking

Docking the MD refined structures of peptide P to H1N1 (PDB Code: 1RUZ) [44] was performed in AutoDock 4.2 [45]. Gasteiger charges were used and nonpolar hydrogens of the macromolecule and ligand were merged. A grid box with the dimensions of 60 Å × 60 Å × 60 Å and a grid spacing of 0.375 Å was set up and centered at the geometric center of the binding box defined with the bound ligand in the crystal structure. Docking was performed by using a Lamarckian genetic algorithm (LGA), with the receptor treated as a rigid body. The docking results were analyzed by AutoDock Tools. The produced conformations were selected based the docking score and manual analysis.

### 3.11. Cytotoxicity Test by MTT Assay

The MTT assay was used to determine the effect of the peptide on the viability of MDCK cells [5,46]. The 96-well plates were used to culture the MDCK cells. After adding 10 μL of PBS containing MTT (0.5 mg/mL) into each well, the solution was incubated at 37 °C for 4 h. After removing the supernatant, 200 μL of DMSO was added into each well. The optical density (OD) for each well was determined at 570 nm. The IC_50_ was calculated as the concentration of the sample at which the number of viable cells was decreased to 50% of that in the cell control.

### 3.12. Cytopathic Effect (CPE) Reduction Assay

The cytopathic effect (CPE) reduction assay was performed according to the previous method [5,47]. MDCK cells were firstly infected with influenza A H1N1 virus (IAV) (MOI = 0.1) and then treated with different concentrations of peptide in triplicate after removing the virus inoculum. After 48-h incubation, 4% formaldehyde was used to fix the cells for 20 min. After removing formaldehyde, 0.1% crystal violet was used to stain the cells for 30 min. After elution of the dye with methanol, the intensity of crystal violet staining for each well was measured at 570 nm.

### 3.13. Acting Mode of Inhibitory Peptide

In order to investigate the inhibitory effects of the peptide on the influenza at different stages of replication, time course analysis was determined according to the previous method [5]. In the pretreatment assay, influenza A virus (IAV, MOI = 3.0) was pretreated with 250 μg/mL of the peptide at 37 °C for 1 h before infection. Then, the virus/peptide mixture was added to MDCK cells for 1 h at 4 °C, and the culture solutions were removed and replaced by sample-free solutions. In the adsorption assay, MDCK cells were infected in solutions containing 250 μg/mL of the peptide at 4 °C after 1-h adsorption and then the culture solutions were removed and replaced by sample-free solutions. In the post-adsorption assay, the virus suspension was added into each well containing a confluent MDCK cell monolayer and then the cells were treated with 250 μg/mL of the peptide after removing the virus inoculums. At 24 h, the antiviral activity was detected by the CPE inhibition assay as described above.

### 3.14. HA Assay

The hemagglutination (HA) assay was performed as previously reported [48]. The virus solutions (10^5^ PFU/mL) were serially diluted in 96-well plates, followed by adding different dilutions of NA-inhibitory peptides. The same volume of 1% standardized chicken red blood cells (cRBCs) prepared according to the World Health Organization (WHO) manuals were added to each well. After 60-min incubation at 4 °C, RBCs in negative wells were sedimented to form red buttons, whereas positive wells had an opaque appearance without sedimentation. HA titers were given as hemagglutination units/50 μL (HAU/50 μL).

### 3.15. Simulated Digestion Assay

Simulated digestion assay was performed according to the previous method with slight modifications [49]. The NA-inhibitory peptide was dissolved in deionized water to prepare 10 mg/mL solution. The pH was adjusted to 2.0 with 1 mol/L HCl and then pepsin (2.86% of the substrate, dry basis) was added. Digestion was performed at 37 °C for 2 h, followed by boiling for 10 min to stop the enzyme activation. Subsequently, the pH was adjusted to 7.5 with 0.2 mol/L NaOH. After adding chymotrypsin (4.00% of substrate, dry basis), the reaction was carried out at 37 °C for 1 h. The sample was submerged in a 95 °C water bath for 10 min to terminate the enzymatic digestion and cooled on ice to room temperature. Then, trypsin (4.00% of substrate, dry basis) was added, and the above steps were repeated. Afterwards, the digested mixtures were centrifuged at 10,000× *g* for 10 min to obtain the digestive juices. The NA inhibition assay in digestive juices was detected as described above.

### 3.16. Statistical Analysis

The data were expressed as mean ± standard deviation (SD). The results were validated by one-way analysis of variance (ANOVA). Duncan’s multiple range tests were performed to determine the differences between means (significance level was set at 5%) using SPSS 20.0 (SPSS Inc., Chicago, IL, USA).

## 4. Conclusions

The novel NA-inhibitory peptide PGEKGPSGEAGTAGPPGTPGPQGL was first prepared from cod skin hydrolysates. Docking studies suggested that hydrogen binding might be the driving force for the binding affinity of the peptide to NA. Time-course analysis showed that the peptide inhibited influenza virus in the early stage of the infectious cycle. The assay of virus titers indicated that the NA-inhibitory peptide could directly affect the virus toxicity and the adsorption by host cells, further proving that the peptide had an anti-viral effect with multiple target sites. The activity of NA-inhibitory peptide was almost inactivated during the simulated in vitro gastrointestinal digestion, suggesting that oral administration was not recommended and the need to further explore other routes of entry. Furthermore, the antiviral activities of peptide against H1N1 in vivo need to be further investigated in order to facilitate the development of peptide in preventing influenza virus infection. The peptide exhibits potential utility in the control of influenza virus infections. This study provides a theoretical basis for guiding the discovery of new natural anti-influenza drugs.

## Figures and Tables

**Table 1:** Purification procedure of NA-inhibitory peptides.

Components	Purification	IC_50_ (mg/mL)	Purification Fold
Hydrolysates (<5000 Da)	Ultrafiltration	6.40 ± 0.13 ^a^	1.00
D	Sephadex G-15	3.50 ± 0.11 ^b^	1.83
D1	RP-HPLC	0.89 ± 0.07 ^c^	7.19

Mean ± standard deviation (SD) (*n* = 3). Values with different superscript letters are significantly different (*p* < 0.05).
